# Multiparametric magnetic resonance imaging for radiation therapy response monitoring in soft tissue sarcomas: a histology and MRI co-registration algorithm

**DOI:** 10.7150/thno.81938

**Published:** 2023-03-05

**Authors:** Matthias Jung, Balazs Bogner, Thierno D. Diallo, Suam Kim, Philipp Arnold, Hannah Füllgraf, Konrad Kurowski, Peter Bronsert, Pia M. Jungmann, Jurij Kiefer, Daniel Kraus, Philipp Rovedo, Marco Reisert, Steffen U. Eisenhardt, Fabian Bamberg, Matthias Benndorf, Alexander Runkel

**Affiliations:** 1 Department of Diagnostic and Interventional Radiology, University Medical Center Freiburg, Faculty of Medicine, University of Freiburg, Germany; 2 Berta-Ottenstein-Programme, University of Freiburg, Faculty of Medicine, Freiburg, Germany; 3 Institute of Surgical Pathology, University Medical Center Freiburg, Faculty of Medicine, University of Freiburg, Germany; 4 Tumorbank Comprehensive Cancer Center Freiburg, University Medical Center Freiburg, Faculty of Medicine, University of Freiburg, Germany; 5 Core Facility for Histopathology and Digital Pathology, University Medical Center Freiburg, Faculty of Medicine, University of Freiburg, Germany; 6 Department of Plastic and Hand Surgery, University Medical Center Freiburg, Faculty of Medicine, University of Freiburg, Germany; 7 Medical Physics, Department of Diagnostic and Interventional Radiology, University Medical Center Freiburg, Faculty of Medicine, University of Freiburg, Germany; 8 Department of Stereotactic and Functional Neurosurgery, University Medical Center Freiburg, Faculty of Medicine, University of Freiburg, Germany

## Abstract

**Rationale:** To establish a spatially exact co-registration procedure between *in vivo* multiparametric magnetic resonance imaging (mpMRI) and (immuno)histopathology of soft tissue sarcomas (STS) to identify imaging parameters that reflect radiation therapy response of STS.

**Methods:** The mpMRI-Protocol included diffusion-weighted (DWI), intravoxel-incoherent motion (IVIM), and dynamic contrast-enhancing (DCE) imaging. The resection specimen was embedded in 6.5% agarose after initial fixation in formalin. To ensure identical alignment of histopathological sectioning and *in vivo* imaging, an *ex vivo* MRI scan of the specimen was rigidly co-registered with the *in vivo* mpMRI. The deviating angulation of the specimen to the *in vivo* location of the tumor was determined. The agarose block was trimmed accordingly. A second *ex vivo* MRI in a dedicated localizer with a 4 mm grid was performed, which was matched to a custom-built sectioning machine. Microtomy sections were stained with hematoxylin and eosin. Immunohistochemical staining was performed with anti-ALDH1A1 antibodies as a radioresistance and anti-MIB1 antibodies as a proliferation marker. Fusion of the digitized microtomy sections with the *in vivo* mpMRI was accomplished through nonrigid co-registration to the *in vivo* mpMRI. Co-registration accuracy was qualitatively assessed by visual assessment and quantitatively evaluated by computing target registration errors (TRE).

**Results:** The study sample comprised nine tumor sections from three STS patients. Visual assessment after nonrigid co-registration showed a strong morphological correlation of the histopathological specimens with *ex vivo* MRI and *in vivo* mpMRI after neoadjuvant radiation therapy. Quantitative assessment of the co-registration procedure using TRE analysis of different pairs of pathology and MRI sections revealed highly accurate structural alignment, with a total median TRE of 2.25 mm (histology - *ex vivo* MRI), 2.22 mm (histology - *in vivo* mpMRI), and 2.02 mm (*ex vivo* MRI - *in vivo* mpMRI). There was no significant difference between TREs of the different pairs of sections or caudal, middle, and cranial tumor parts, respectively.

**Conclusion:** Our initial results show a promising approach to obtaining accurate co-registration between histopathology and *in vivo* MRI for STS. In a larger cohort of patients, the method established here will enable the prospective identification and validation of *in vivo* imaging biomarkers for radiation therapy response prediction and monitoring in STS patients via precise molecular and cellular correlation.

## Introduction

Soft tissue sarcomas (STS) are a rare and heterogeneous group of mesenchymal-derived malignant tumors representing approximately 1% of all malignancies.^1^ The accepted standard treatment strategy for localized STS includes (neo)adjuvant radiation therapy followed by wide resection.^2^-^6^ The extent to which neoadjuvant radiation therapy influences the outcome of STS has yet to be studied systematically. Furthermore, no reliable methods for peritherapeutic monitoring of tumor viability have been established, and imaging has not been integrated into the preoperative course in a structured manner.

To date, retrospective, postoperative estimation of the cell necrosis rate by histopathological assessment is the only objective criterion for evaluating preoperative treatment response.^7^, ^8^ The histopathological evaluation is challenging due to the substantial intratumoral STS heterogeneity and requires thorough sampling to avoid misdiagnosis. Magnetic resonance imaging (MRI) allows a noninvasive assessment of the entire tumor. A multiparametric MRI approach can characterize distinct tumor components and quantitatively assess changes within these components as the tumor responds to radiation therapy. The use of diffusion-weighted imaging (DWI) to assess tumor cellularity and dynamic contrast-enhanced (DCE) sequences to assess tumor vascularity increases the sensitivity of MRI in evaluating response to neoadjuvant radiation therapy.^9^-^11^ Intravoxel incoherent motion (IVIM) provides information about tissue microcirculation without the need for contrast agents and has demonstrated a potential for differential diagnosis of malignant and benign tumors and therapy monitoring.^12^ However, the diagnostic accuracy of new imaging parameters requires validation, preferably by histopathological verification.

Multiple methods for co-registration of mpMRI with histopathology have been described for prostate cancer.^13^, ^14^ To date, no co-registration method exists for the correlation of histopathology with MRI findings of STS. Previous studies have used simple techniques to correlate MRI parameters with pathological findings. Only a few exemplary regions of interest (ROI) or a pathological classification system for the entire surgical specimen were considered and assumed to represent the entire tumor mass.^9^-^11^, ^15^ These methods do not allow a sufficiently differentiated analysis of the complex response of different portions of the heterogeneous tumor mass to radiation therapy.

Therefore, this study aimed to develop a co-registration procedure between (immuno)histopathology and *in vivo* mpMRI that enables the identification of imaging biomarkers to characterize the response of STS to radiation therapy.

## Materials and Methods

### Subjects

The local Institutional Review Board approved the study (EKFR 21-1735). All procedures performed involving human participants were in accordance with the ethical standards of the institutional and/or national research committee and with the 1964 Helsinki declaration and its subsequent amendments or comparable ethical standards. Inclusion criteria were histologically confirmed Grade II or III STS, planned to be treated with neoadjuvant radiation therapy and wide resection, written informed consent, and age > 18 years. Exclusion criteria were MRI contraindications (claustrophobia, pregnancy, non-MRI-safe implants), metastatic disease, and creatinine clearance < 30 ml/min. Without pre-selection, the first three eligible patients were included in this study regardless of the entity or configuration of the tumor. Informed written consent was obtained from all three patients scheduled for neoadjuvant radiation therapy and STS resection.

### Co-registration Process

**Figure 1:**
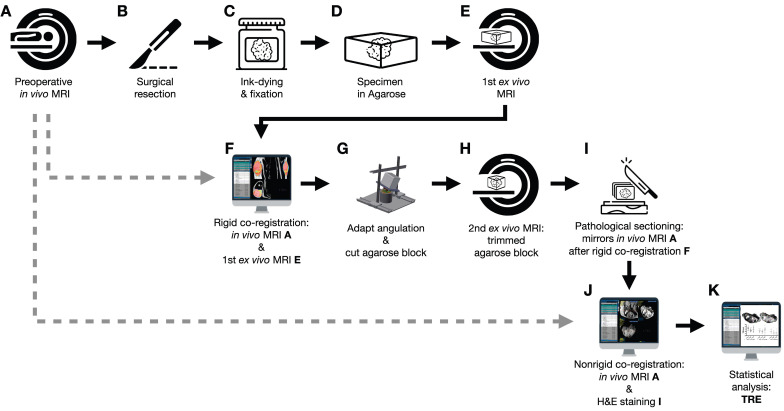
Flowchart of the co-registration procedure.

Figure 1 shows an overview of the co-registration process described below.

### *In vivo* mpMRI

The mean days between the last preoperative mpMRI (Figure 1A) and surgery were 6.7 days. MR imaging was performed on a 3.0 T MR scanner (Vida, Siemens Healthineers) using a dedicated 18-channel body coil (Siemens Healthineers). MR parameters are shown in Table 1. After axial and coronal T1- and T2-weighted (T2w) anatomic images, quantitative MRI sequences were acquired in axial orientation identical to the planning of the T2w sequence: diffusion-weighted imaging (DWI) for the apparent diffusion coefficient (ADC), intra-voxel incoherent motion (IVIM) for pseudo diffusion coefficient D*, perfusion fraction *f*, blood flow-related parameter fD*, and dynamic contrast-enhanced (DCE) MRI for *K^trans^*, *v_e_*, *k_ep_*, *v_p_*. Only the T2w sequence was used for the co-registration process described below. The final co-registration, however, is valid for the entire mpMRI because of the identical planning and sectioning of the T2w sequence and the quantitative sequences. Detailed MR parameters are shown in Table 1.

### First *ex vivo* MRI

Following surgery (Figure 1B), the specimens were ink-dyed in a standardized procedure (Figure 1C). Depending on the tumor size, the specimen was fixed with buffered formalin for 48-72 hours (Figure 1C). After formalin fixation, the specimen was embedded in 6.5% agarose (Figure 1D). Subsequently, an *ex vivo* MRI of the agarose block was performed (Magnetom Trio Tim, Siemens Healthineers, axial T2-weighted images, 2 mm slice thickness; Figure 1E).

### Rigid *in vivo* and *ex vivo* MRI co-registration

Since the orientation of the specimen in the agarose block and the axial sectioning of the first *ex vivo* MRI is random, a rigid *in vivo* and* ex vivo* MRI co-registration was performed on the nora medical imaging platform using the built-in “Navigation” tool (available free of charge online: http://ukl-nora-demo.ukl.uni-freiburg.de/nora/index.php?viewer; Figure 1F): *In vivo* axial T2w MRI slices were used as the fixed reference images. The *ex vivo* MRI was defined as the “moving image” and manually registered to the *in vivo* MRI using a three-dimensional (3D) rigid registration, i.e., translation and rotation.

### Specimen angulation and agarose-block trimming

The resulting rotation matrix was stored as a json file to calculate the three necessary input angles for subsequent orientation of the custom-built angle plate (Supplemental Figure 1; Standard for the exchange of product model data (STEP) file is provided as a supplemental file online).

The rotation matrix (json file) was virtually applied to the coordinate systems of the custom-built angle plate, and the agarose embedded tumor on top of it in a custom MATLAB script (The MathWorks, Inc., Matlab script provided as a supplemental file online). An additional correction was added if the original *in vivo* scan was angulated. Using Rodrigues' rotation formula 16, a virtual back-rotation was performed to obtain the two planar angles determining the elevation and the azimuthal angle. The angular plate was then manually rotated to the calculated angles. The agarose block was placed on top and trimmed on all six sides in the respective planes of the world coordinate system (Figure 1G). After cutting, the orientation of the tumor specimen in the agarose block was aligned with the former *in vivo* orientation of the tumor in the patient.

### Second *ex vivo* MRI

The trimmed agarose block was placed in a dedicated localizer with a 4 mm measuring grid attached to the sidewalls and visible on MRI as a negative in the agarose block (Supplemental Figure 2; STEP file is provided as a supplemental file online). In this localizer, a second *ex vivo* MRI of the fitted agarose block was performed (Magnetom Trio Tim, Siemens Healthineers, axial T2-weighted images, 4 mm slice thickness; Figure 1H). Axial, sagittal, and coronal localizers were used for positioning the MRI slices. All of the second *ex vivo* MRI slices were planned following the 4mm grid attached to the sidewall.

### Histopathology preparations

A custom cutting device was used to cut sections every 4 mm corresponding to the MRI localizer of the second *ex vivo* MRI (Supplemental Figure 3, STEP file is provided as a supplemental file online; Figure 1I). This guaranteed equal cutting angles between the tumor specimen and the second *ex vivo* MRI slices and a correct localization for cutting the specimen according to the second *ex vivo* MRI. An experienced pathologist performed sectioning. To optimize the alignment of mpMRI and histology, large area sections were prepared for subsequent microtomy. These are specifically processed macroscopically and histologically and cover twice the area of a standard slide. Possible distortion artifacts by microtomy are thus minimized. Immunohistochemical staining was performed using anti-ALDH1A1 and anti-MIB1 antibodies. For this purpose, the 2 μm thick large area sections were deparaffinized and underwent heat-induced antigen retrieval. The large-area slices were then incubated with the respective primary antibody (ALDH1A1 as radioresistance and EMT-/MIB1 as proliferation marker) and with a detection system. All sections were counterstained with hematoxylin and eosin (H&E), digitized, and annotated.

### Nonrigid histopathology and MRI co-registration

Co-registration of macroscopic and histopathological slices to second *ex vivo* MRI and *in vivo* mpMRI was performed using the navigation tool of the nora - medical imaging platform (Figure 1J). In this step, macroscopic and histopathological slices were digitized and transferred to the nora - medical imaging platform.

Corresponding landmarks were manually identified across the macroscopic, histopathological, *in vivo,* and *ex vivo* MRI sections. In consensus, a pathologist (8 years of experience) and a radiologist (5 years of experience) defined a total of n=30-40 landmarks for every pair of MRI and pathology sections, corresponding to contours of marked tumor portions, such as solid/cystic/necrotic parts. Subsequently, the landmarks were randomly split into two independent sets, one for nonrigid elastic co-registration and the other for evaluating the accuracy of the registration. To avoid errors due to the random split of landmarks, this step was iteratively repeated 100 times per pair of sections.

### Assessment of co-registration accuracy

**Figure 2:**
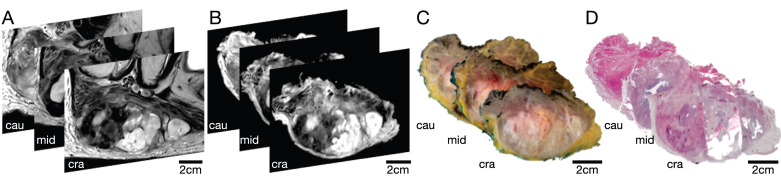
Selection of 3 representative STS sections (cranial, middle, and caudal part) of patient 1. (A) *In vivo* MRI; (B) *Ex vivo* MRI; (C) Macropathological specimen; (D) Histopathological H&E staining. Cau: caudal; cra: cranial; mid: middle.

**Figure 3:**
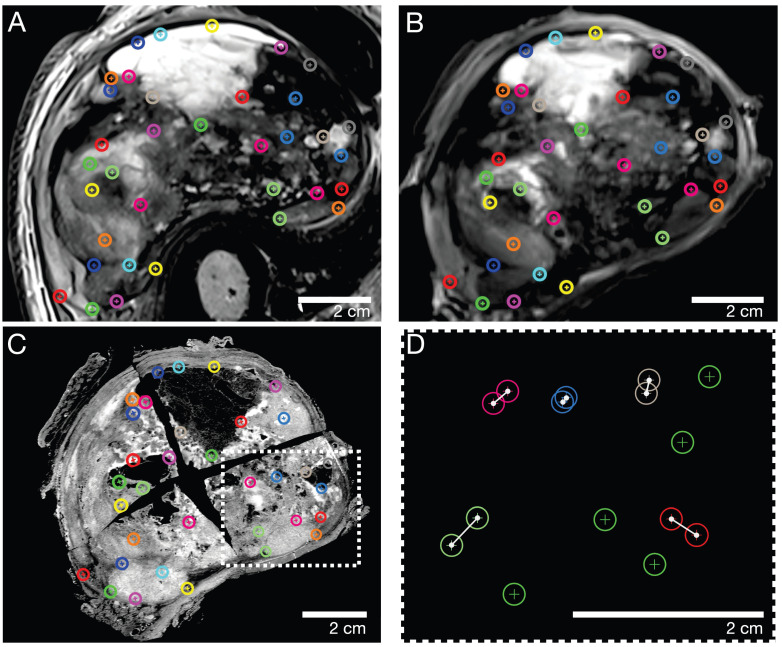
Representative caudal section of a pleomorphic sarcoma (patient 2). Color-coded landmarks were defined by a pathologist and radiologist on (A) *in vivo* MRI, (B) *ex vivo* MRI, the specimen (not shown), and (C) the H&E microtomy section. (D) Exemplary illustration of the TRE computation: landmarks were randomly split into two sets, one for nonrigid co-registration (green) and the other for evaluating the co-registration accuracy as the Euclidian distance in millimeters (white connections of landmarks). TRE: target registration error

To assess the co-registration accuracy, three representative sections (proximal, middle, and distal parts of the STS) of each study participant were analyzed. The special 4mm grid MRI localizer (Supplemental Figure 2), adapted to the cutting device (Supplemental Figure 3), allows an easy matching of the pathology slices to the corresponding MRI slices (Figure 2). The registration process results were first qualitatively assessed by visual assessment of the contours in terms of plausible deformations. For quantitative evaluation of the accuracy, target registration error (TRE) was measured as the Euclidean distance between each corresponding anatomic landmark annotated in consensus (Figure 3). For each iteration of elastic co-registration, the landmarks used to evaluate the TRE were independent of the landmarks used for initial registration. TRE for each pair of sections: histology and *ex vivo* MRI, histology and *in vivo* MRI, and *ex vivo* MRI and *in vivo* MRI are reported as the median and interquartile range (IQR) in millimetres (mm). Due to the slightly right-sided skewed data distribution, nonparametric tests were used for statistical analysis. Kruskal-Wallis test was used for comparisons of more than two groups. For pairwise group comparisons, Bonferroni corrected Wilcoxon rank-sum test was performed to avoid alpha error accumulation. Statistical significance was indicated by p-values < 0.05. Statistical analysis was performed using R V4.2.2 (R Core Team, www.r-project.org, 2022).

### Statistical clustering and mpMRI - Histology comparison

The quantitative map values of the DWI (i.e. ADC), IVIM (i.e. f, D*, and fD*), and DCE (i.e. *K^trans^*, *v_e_*, *k_ep_*, *v_p_*) sequence were extracted for each voxel within the tumor ROI of the cranial section of patient 1. Parametric map values within the tumor ROI were equally weighted for subsequent classification into four groups, based on histological analysis. We performed K-means clustering (Python machine learning library scikit-learn 17) to split the voxels of the tumor ROI into a set of 4 groups. The classification and voxel position identified by clustering were mapped onto the T2 sequence by color coding. Clustering was performed using only parametric mpMRI-values. Spatial information was not used as an additional input, but only to identify the clusters on the T2 image. Four representative ROIs - one per cluster - were defined on the co-registered histology and mpMRI section. One-way ANOVA with post hoc pairwise t-tests (Bonferroni-Holm adjusted) was performed to compare differences in mpMRI parameters. Statistical significance was indicated by p-values < 0.05. Statistical analysis was performed using R V4.2.2 (R Core Team, www.r-project.org, 2022).

## Results

### Study participants

**Figure 4:**
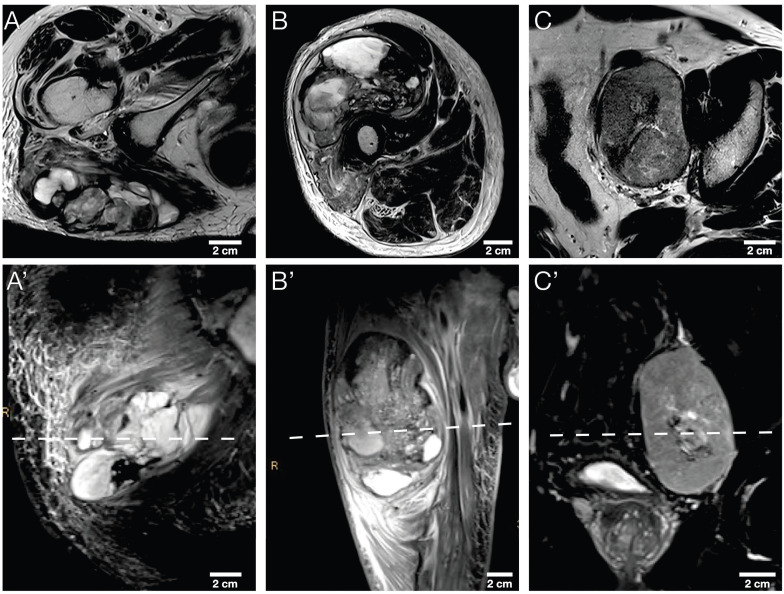
Morphological MRI sequences of the three STS patients. Exemplary axial T2w images (A, B, C) and corresponding coronal T2w-STIR images (A', B', C') of the three STS patients included. The dashed line in the lower row indicates the sectioning plane of axial T2w images in the upper row. STIR, short tau inversion recovery.

The study sample comprised nine tumor sections, three representative tumor slides from a total of three STS patients: A section of each cranial, middle, and caudal tumor part. More detailed patient characteristics are provided in Table 2. Exemplary axial T2w and coronary T2w-STIR *in vivo* MR images of the three STS patients are illustrated in Figure 4.

### Qualitative visual assessment

**Figure 5:**
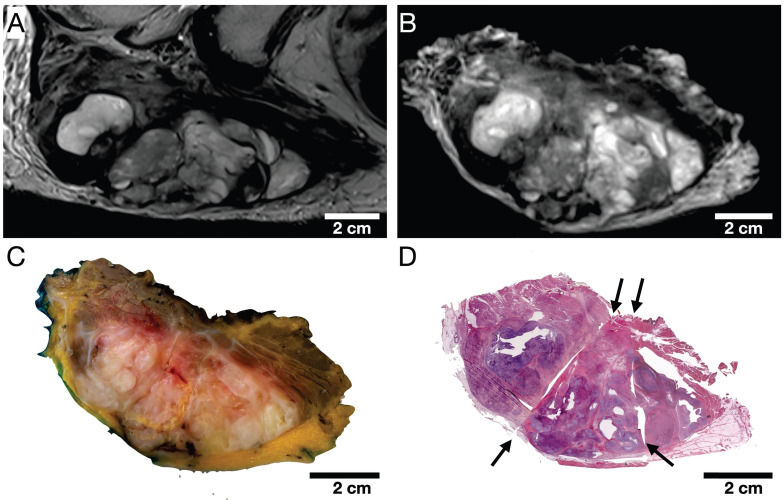
Representative cranial section of an MFS (patient 1)**.** Visual assessment of (A) axial *in vivo* T2w, (B) axial *ex vivo* T2w, (C) macroscopic section, and (D) H&E stained microtomy section shows a high spatial and morphological correlation. Note the light deformations of the microtomy section (D) at the cutting edges (black arrows) as well as split and microtomy artifacts (white arrows).

The qualitative visual assessment showed a high spatial and morphological correlation of histopathological specimens with *in vivo* mpMRI after neoadjuvant radiation therapy (Figure 5). Using a custom-built 4mm grid MRI localizer matched to the cutting device, the corresponding MRI and pathology slices could easily be identified. Due to the relatively large size of the STS, the macroscopic tissue sections (Figure 5C) were divided into 2-4 parts (Figure 5D) to fit into the large area sections for further histological processing. In addition to prefixation (split) and tissue processing (microtomy) artifacts of histological processing, we observed deformations, particularly at these cutting edges (Figure 5D).

### Target registration error analysis

**Figure 6:**
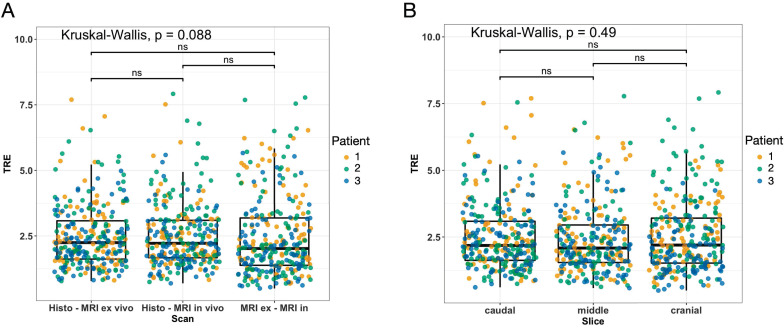
Results of the target registration error analysis illustrated as boxplots combined with jitter plots. (A) Pairs of MRI and histology sections: left, histology - *ex vivo* MRI; middle, histology - *in vivo* MRI; right, *ex vivo* - *in vivo* MRI. (B) Sections of the caudal (left), middle (middle), and cranial (right) STS parts. Boxplots: middle line represents the median; the upper and lower ends of the box represent the 75th and 25th percentiles, respectively. Jitter plot: Dots indicate landmarks color-coded for each patient. Y-axis: target registration error (TRE) in mm.

 The overall median TRE for the nonrigid co-registrations was 2.25 mm (IQR 1.46 mm) for the fusion of the microtomy section to the *ex vivo* MRI, 2.22 mm (IQR 1.41 mm) for the fusion of the microtomy section to the *in vivo* mpMRI, and 2.02 (IQR 1.79 mm) for the fusion of the *ex vivo* MRI to the *in vivo* mpMRI (Figure 6A). The mean TRE per STS volume for the fusion of the microtomy section to the *in vivo* mpMRI was 0.009 ± 0.004 mm/mL (Table 2). Patient 1 showed the widest range of distinct heterogeneous tumor volumes (range 0.8 to 65.6 mL) and axial diameters (range 10.2 to 39.9 mm) of all included patients with a TRE per STS volume of 0.013 mm/mL (Supplemental Figure 4).

There were no statistically significant differences in TRE for the three pairs of sections (all p > 0.05; Figure 6A). Additionally, there were no differences in TRE in pairwise comparisons of the overall TRE for caudal (median 2.18 mm; IQR 1.46 mm), middle (median 2.09 mm; IQR 1.40 mm), and cranial (median 2.20 mm; IQR 1.68 mm) STS sections (all p > 0.05; Figure 6B).

### Statistical clustering and initial quantitative results

**Figure 7:**
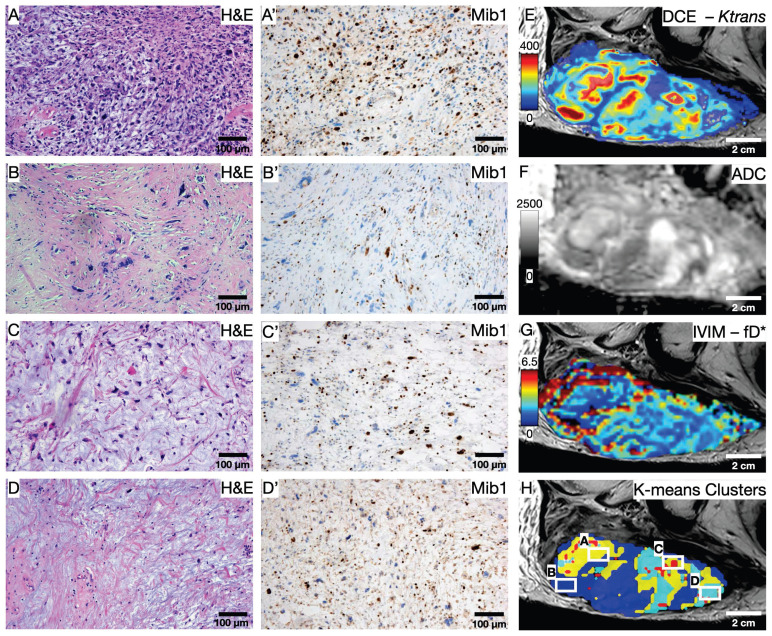
Initial comparison between (immuno)histology and quantitative mpMRI parameters. (A-D) H&E and (A'-D') Mib1 stainings in 100x magnification show the four predominant histology patterns on the section: Cell-rich - vital tumor cells (A and A'); sclerotic background - vital tumor cells (B and B'); myxoid - vital tumor cells (C and C'); predominant myxoid - single vital tumor cells (D and D'). (E-H) Exemplary parametric maps for each quantitative MRI sequence: DCE - *K^trans^* (E); DWI - ADC (F); IVIM - fD* (G). (H) Result of the K-means clustering based on all 8 available parametric Maps; White squares indicate the area of the histological images.

**Figure 8:**
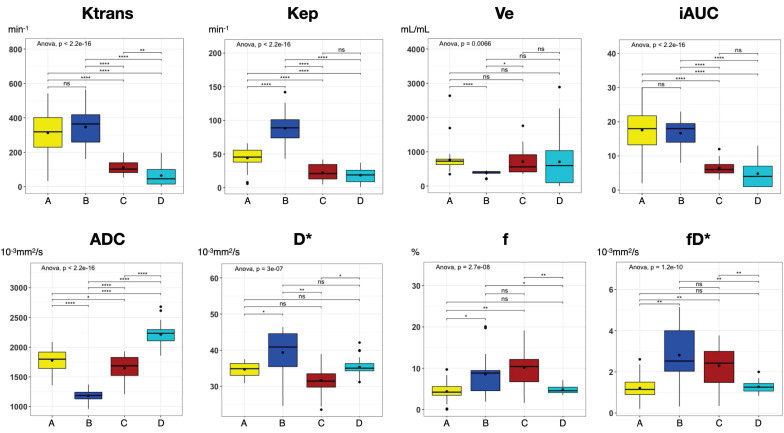
Boxplots of all parametric MRI parameters for each distinct Region A-D as shown in Figure 7. Boxplots: middle line represents the median; the upper and lower ends of the box represent the 75th and 25th percentiles, respectively. Black dot in Box represents the mean. Color-coding identical to Figure 7: region A, yellow; region B blue; region C, red, region D, cyan. P values were obtained using one-way ANOVA, followed by the t-tests (Bonferroni-Holm adjusted) for comparison of mean values between regions. *, p < 0.05; **, p < 0.01; ***, p < 0.001; ****, p < 0.0001; ns, not significant.

 To illustrate the usefulness of the presented co-registration method, we exemplarily performed an initial comparison between (immuno)histology (Figure 7A-D') and quantitative mpMRI parameters (Figure 7E-G) in the cranial section of patient 1. K-means clustering based on all available mpMRI parametric maps (*K^trans^*, *v_e_*, *k_ep_*, *v_p_*, ADC, D*, f, fD*; Figure 7H) showed robust discrimination of the four distinct histological patterns on the section: Cell-rich - vital tumor cells (Figure 7A and A'); sclerotic background - vital tumor cells (Figure 7B and B'); myxoid - vital tumor cells (Figure 7C and C'); predominant myxoid - single vital tumor cells (Figure 7D and D'). An overview of all parameters is provided in Table 3. In post-hoc pairwise comparisons of the different regions, we found statistically significant differences for all parametric MRI maps (Figure 8).

## Discussion

In this study, we report the results of a co-registration algorithm for spatial correlation of (immuno)histopathology and *in vivo* mpMRI of STS. In our procedure, highly accurate registration of histopathology to preoperative *in vivo* mpMRI is achieved via an intermediate registration of *in vivo* mpMRI to an additionally acquired *ex vivo* MRI. An initial rigid 3D registration of the first *ex vivo* MRI with the *in vivo* mpMRI is performed before the preparation of the pathological sections. This first registration step ensures that the orientation of the specimen in the agarose block corresponds to the former orientation of the tumor in the patient. Subsequently, a second *ex vivo* MRI of the fitted agarose block is performed in a custom-built localizer, which defines the sectioning of the pathological slides, guaranteeing equal cutting angles and positions between the tumor specimen and the second *ex vivo* MRI slices that already reproduced the slice plane of the *in vivo* mpMRI scan.

To date, the postoperative histopathological examination is the only objective criterion for assessing preoperative treatment-related response to neoadjuvant radiation therapy. Conventional preoperative MRI parameters such as tumor volume reduction or cystic degeneration, presumed to indicate tumor necrosis, have shown limited value in evaluating treatment response in STS.^9^, ^18^, ^19^ This emphasizes an emerging need for advanced imaging parameters reflecting the STS response to radiation therapy. Functional MRI sequences, including DCE, DWI, and IVIM have shown potential for noninvasive therapy monitoring.^9^-^11^ However, new imaging parameters and their diagnostic and predictive accuracy require histopathological validation. This is preferably performed by directly comparing MRI with whole-mount histopathology of the surgical specimen using imaging registration. Previous studies that analyzed radiation therapy response in STS using mpMRI did not perform a slice-to-slice comparison of histopathology and *in vivo* MRI. In the study by Soldatos et al., the pathological assessment was based on a semiquantitative score for the entire tumor. Percentages of viable tumor, treatment-related necrosis, and posttreatment granulation tissue and fibrosis were determined on each glass slide and subsequently averaged, resulting in an estimated score for each specimen.^9^ Similarly, an estimated amount of necrosis and viable tumor with an additional dichotomization in optimal (≥95% necrosis) and suboptimal (<95% necrosis) treatment response on the whole specimen was used by Huang et al.^11^ In the study by Winfield et al., pathological sectioning was based on a free-hand estimation of the tumor orientation, derived from a single axial T2-weighted MRI slice. Up to three ~1cm^2^ ROIs per tumor were then chosen to represent the entire specimen.^10^ In contrast, our approach allows the assessment of, e.g., vascularization and cell density in the whole tumor before and after radiation therapy, with excellent spatial accuracy.

For prostate cancer, several sophisticated co-registration methods have been described. To the best of our knowledge, this is the first study to describe a comparable co-registration algorithm for STS. However, the co-registration of histological images and MRI is challenging. The methods described previously for prostate cancer vary from simple visual alignments to rigid and nonrigid computer-based registrations.^20^-^25^ Results of the registration process are affected by specimen deformations due to formalin fixation and in-plane or out-of-plane registration mismatches. To reduce these errors, intermediate registration with *ex vivo* images of the specimen was introduced.^24^, ^26^-^28^ Consequently, pathological sectioning resembled the *ex vivo* MRI plane but not necessarily the *in vivo* imaging plane. Pathological sections should be performed in the same plane as *in vivo* MRI slicing. A common method to address this issue for prostate specimens is to use a device or 3D-printed mold that fixes the specimen in the desired orientation.^29^, ^30^ The primary goal of radical prostatectomy is to remove the entire prostate, including the capsule. A 3D-printed mold can be easily adapted to such a sharply bordered specimen. In contrast, STS are often significantly larger, and the specimen shape is defined by the irregular margins of soft tissues. To compensate for this, in the procedure presented here, the surgical specimen is fixated in agarose. Via the initial co-registration of a first *ex vivo* MRI of the agarose block with the *in vivo* mpMRI, we solved this potential problem of deviating cross-sectioning planes. To match each pathological slide to the corresponding *in vivo* mpMRI slide, a second *ex vivo* MRI of the fitted agarose block was performed on a custom localizer with a 4mm grid attached to the side walls. To our knowledge, this is the first co-registration method that uses two separate *ex vivo* MRIs for intermediate *ex vivo* to *in vivo* MRI co-registration. This two-step intermediate co-registration ensured identical orientation of the pathological sectioning with the *in vivo* MRI and subsequently allowed straightforward identification of the corresponding pathological and *in vivo* MRI slides.

To give a more intuitive scale to our results and make them comparable to other studies, we introduced a ratio of TRE in mm and tumor volume in mL. Our novel co-registration procedure resulted in a mean TRE per STS volume of 0.009 mm/mL. Considering the smallest identifiable tumor region with an axial diameter of 10.2 mm and a volume of 0.8 mL in Patient 1, all tumor regions could reliably be identified regardless of size, volume, entity, and heterogeneity in our study. Several previous studies reported TRE of co-registration methods of *in vivo* MRI with histopathology performed on the brain 31-34 and the prostate 27, 35, with only a few studies examining other organs such as the colon 36 or the larynx 37. Four of these previous studies reported TRE together with the respective tumor or organ volumes: Taking the reported TRE and volumes of tumor or organ specimens, these procedures would result in a TRE per volume ranging from 0.052 ml/mL for a neocortical specimen 34 to 0.28 mm/mL for a prostate specimen.^35^ Comparing our results with the above-mentioned studies, it can be concluded that our novel co-registration method is spatially highly accurate, especially considering the rather large STS included. However, direct comparison of our results with those of other studies is limited to some degree because of the different tumor entities and the different methods used for co-registration of *in vivo* MRI with histopathology and should be interpreted carefully.

Jardim-Perassi et al. 38 were the first to demonstrate the ability to identify different histologically verified tumor characteristics using a clustering approach of quantitative MRI parameters in a breast cancer mouse model. To demonstrate the value of our co-registration procedure, we performed an exemplary first comparison between quantitative *in vivo* mpMRI parameters and (immuno)histology in one patient. Using a similar approach as Jardim-Perassi et al., we performed a machine-learning clustering of all eight available mpMRI parameters based on histological parameters, such as cell density and Mib1 proliferation index. Interestingly, the clustering resulted in robust discrimination of the four distinct histological patterns in this MFS section. These results further support the hypothesis that non-invasive quantitative MRI metrics may be used to identify tumor subregions with different biological behavior in heterogeneous tumors such as STS. In addition, we found significant differences in all parametric MRI maps when analyzing the four histological patterns, suggesting that each individual quantitative mpMRI parameter likely contributed to the clustering. This is consistent with previous studies and highlights the importance of a multiparametric approach to identify differences in histological and biological characteristics of heterogeneous STS tumor parts.^39^, ^40^

Our study has several limitations. First, this pilot study reports the initial results of a newly developed co-registration procedure for STS with only a small sample size of 9 tumor sections from 3 patients. Second, after formalin fixation of the intact specimens (without prior lamination), we noticed a slight concentric shrinkage of the samples. However, no method was applied to estimate the shrinkage percentage and its potential error in the co-registration procedure. As reported above, our approach to compensate for that and through-plane artifacts was to analyze three slides of each patient from a caudal, middle, and cranial tumor part. Given the strong spatial correlations reported here and the fact that no significant differences between the three parts were found, our results indicate that the shrinkage error was marginal and negligible. Third, due to the relatively large size of the STS of our study participants, we used a total of 30-40 landmarks per tumor slice (Figure 3), which may pose a risk of statistical overfitting.

## Conclusion

Here we present the first algorithm for co-registration of histopathology and *in vivo* mpMRI for STS. Our initial results show a promising approach for obtaining highly accurate spatial co-registration of pre- and posttherapeutic MRI with whole-mount histological specimens of STS. In a larger cohort of patients, the method established here will enable the prospective identification and validation of *in vivo* MR imaging biomarkers for radiation therapy response monitoring of STS via precise molecular and cellular correlation.

## Supplementary Material

Supplementary figures and files.

## Figures and Tables

**Table 1:** Multiparametric MRI sequence parameters.

Sequence	T2w TSE STIR	T1w TSE Dixon	T2w TSE	IVIM	DWI	VIBE DCE	T1w TSE Dixon	T1w VIBE Dixon
Additional features	2D	2D	2D	2D	2D	3D	2D	3D
Plane	Cor	Cor	Tra	Tra	Tra	Tra	Cor	Tra
Echo time (TE; ms)	40	10	104	64	61	1.14	9.8	2.46, 3.69
Repetition time (TR; ms)	5600	650	11900	6900	7780	3.02	650	5.5
Field of view (FOV; mm)	500	500	220	220	220	220	500	350
Slice thickness (mm)	4	4	4	4	4	4	4	3
In-plane resolution (mm^2^)	1.0 × 1.0	0.8 × 0.8	0.3 × 0.3	1.8 × 1.8	0.9 x 0.9	1.1 x 1.1	0.9 x 0.9	0.9 x 0.9
Flip angle (°)	140	160	160	90	180	15	90	9
Number of slices	30	30	40	40	40	40	30	112
Slice distance (mm)	4.8	4.8	4	4	4	4	4.8	3
Echo Train Length	12	3	25	53	0	1	3	2
Bandwidth per pixel (Hz)	222	845	200	1812	883	789	868	690
Phase Encoding Direction	Row	Row	Col	Col	Col	Col	Row	Col
Number of averages	1	1	3	3	1	1	2	3
Acquisition time (min)	01:54	01:36	03:48	04:45	08:43	04:33	04:24	03:08
Fat water contrast	Standard	Fast Dixon	Standard	SPAIR	Fat Saturation	Standard	Fast Dixon	Dixon
Fat Saturation	STIR	Strong	-	Strong	Strong	-	Strong	-
Diffusion Mode	-	-	-	3D Diagonal	4-Scan Trace	-	-	-
B value (s/mm^2^)	-	-	-	0, 10, 20, 30, 40, 50, 70, 100, 200, 400, 600, 800	50, 400, 1000	-	-	-
Calculated b value (s/mm^2^)	-	-	-	-	1400	-	-	-
EPI factor	-	-	-	90	95	-	-	-
Contrast enhancement	No	No	No	No	No	Yes	Yes	Yes
Temporal resolution (s)	-	-	-	-	-	5.5	-	-
Measurements	-	-	-	-	-	50	-	-

Col: column; Cor: coronal; DCE: dynamic contrast enhanced imaging; DWI: diffusion weighted imaging; EPI: echo-planar imaging; FOV: field of view; fs: fat-saturated; IVIM: intravoxel incoherent motion; SPAIR: spectral attenuated inversion recovery; STIR: short tau inversion recovery; Tra: transverse; VIBE: volumetric interpolated breath-hold examination; w: weighted

**Table 2:** Patient characteristics.

Patient	1	2	3
**Age**	91y	83y	57y
**Sex**	Female	Male	Male
**STS Type**	MFS	UPS	LMS
**Grading**	G2	G3	G2
**Location**	Right gluteal region	Right upper leg	Left pelvis
Transversal Size* (cm)	10.6 x 3.9	10.2 x 7.1	8.4 x 6.0
Coronal Size* (cm)	11.2 x 6.6	13.8 x 7.1	8.4 x 5.4
**STS Volume (mL)**	165.0	510.5	215.9
**TRE/Volume (mm/mL)**	0.013	0.005	0.009

LMS: leiomyosarcoma; MFS: myxofibrosarcoma; STS: soft tissue sarcoma; UPS: undifferentiated pleomorphic sarcoma; *Maximum size in centimeters (cm) measured on the last preoperative *in vivo* mpMRI after neoadjuvant radiation therapy

**Table 3:** Representative (immuno)histological and mpMRI parameters for each cluster

Region	H&E	Cell density	Mib1	Ktrans	Kep	Ve	iAUC	ADC	D*	f	fD*
A	Cell-rich; vital tumor cells	5361	40.4	313.2 ± 130.9	44.2 ± 15.1	759.8 ± 360.1	17.6 ± 6.7	1776.2 ± 184	34.7 ± 1.9	4.4 ± 2.1	1.2 ± 0.6
B	Sclerotic background; vital tumor cells	5046	17.1	347.3 ± 110.4	88.3 ± 22.6	388.6 ± 61.5	16.7 ± 4.3	1181.7 ± 93.4	39.4 ± 6.5	8.6 ± 5.5	2.8 ± 1.4
C	Myxoid; vital tumor cells	1928	21.8	112.2 ± 42.8	22.3 ± 12.3	710.8 ± 397	6.5 ± 2.3	1647.3 ± 203.6	31.6 ± 4.3	10.1 ± 5	2.3 ± 1
D	Predominant Myxoid; single vital tumor cells	1410	37.3	64.2 ± 61.4	18.6 ± 10.7	705.9 ± 724.7	4.8 ± 4.1	2212.3 ± 188.3	35.4 ± 2.4	4.8 ± 1	1.3 ± 0.3

Cell density, number of cells/mm^2^; Mib1, %; Ktrans, min^-1^; Kep, min^-1^; Ve, mL/mL; iAUC, arbitrary units; ADC, 10^-3^mm^2^/s; D*, 10^-3^mm^2^/s; f, %; fD*, 10^-3^mm^2^/s;

## Abbreviations

Cm: centimeters
Col: column
Cor: coronal
ALDH1A1: aldehyde dehydrogenase 1 family, member A1
DCE: dynamic contrast-enhancing
DWI: diffusion-weighted imaging
EPI: echo-planar imaging
FOV: field of view
fs: fat-saturated
IVIM: intravoxel-incoherent motion
IQR: interquartile range
mm: millimeters
MRI: magnetic resonance imaging
mpMRI: multiparametric magnetic resonance imaging
ROI: region of interest
SPAIR: spectral attenuated inversion recovery
STIR: short tau inversion
STS: soft tissue sarcoma
T2w: T2-weighted
Tra: transverse
TRE: target registration error
VIBE: volumetric interpolated breath-hold examination
