# Supplementary material for: Multiparametric magnetic resonance imaging for radiation therapy response monitoring in soft tissue sarcomas: a histology and MRI co-registration algorithm
Source: Theranostics. 2023 Mar 5;13(5):1594–606. doi: 10.7150/thno.81938 (PMC10086213; doi:10.7150/thno.81938)
Supplement: Supplementary file 1 — Supplementary figures and files. [file thnov13p1594s1.zip › SUPPLEMENTARY MATERIALS.pdf]

SUPPLEMENTARY MATERIALS

Figure 1: Technical Drawing of the custom-built angle plate.

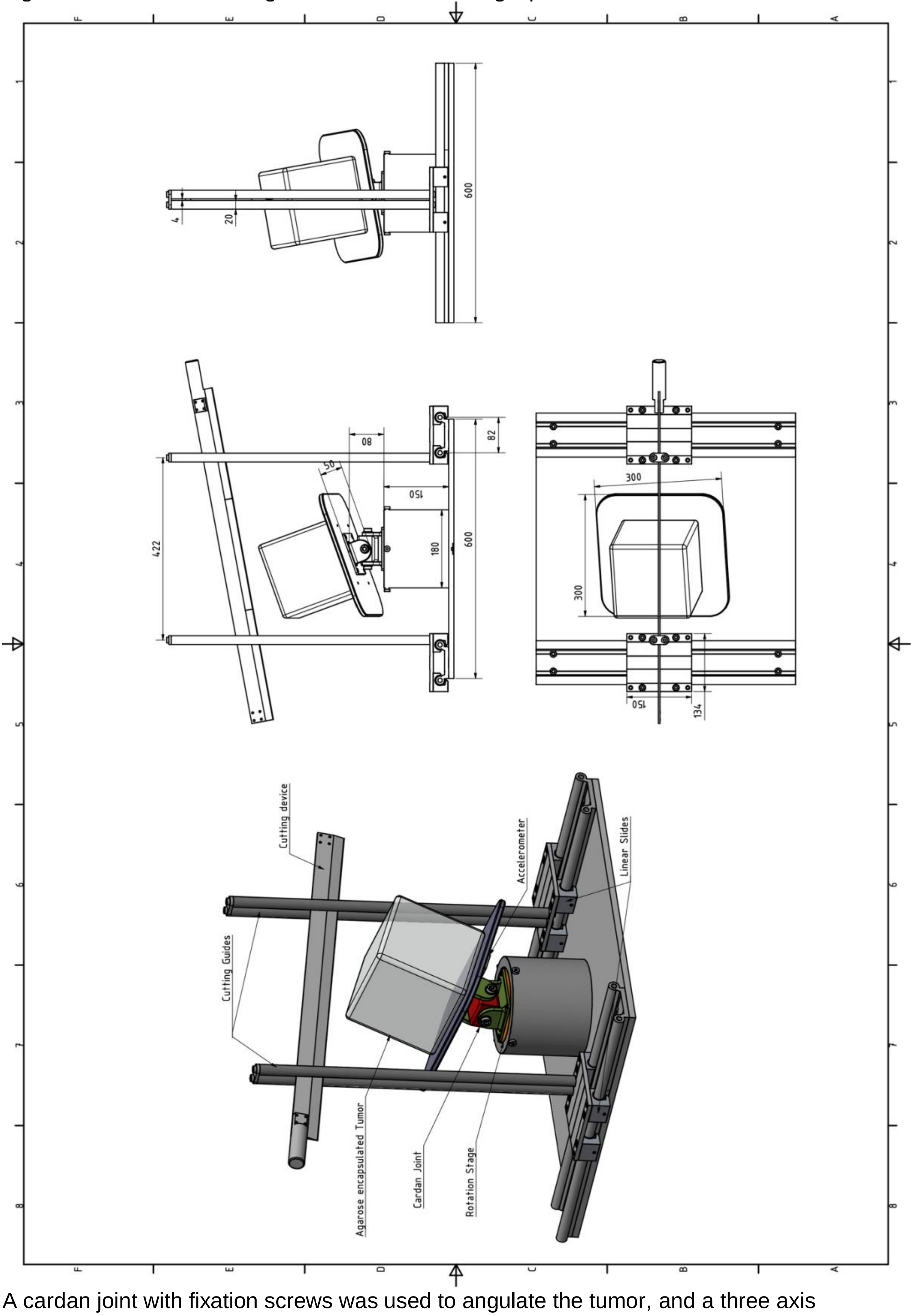

A cardan joint with fixation screws was used to angulate the tumor, and a three axis accelerometer (Pololu LSM6DS33) was mounted below the support panel to measure the two angles towards the xy-plane. A ball bearing enabled the rotation of the angulation stage

and the rotational position was recorded using a rotational encoder (Broadcom AEDM-2000). All data was gathered using a microcontroller (PJRC Teensy 4.1) and then displayed in a Matlab GUI via a serial USB connection.
Standard for the exchange of product model data (STEP) file is provided online as supplemental data.

Figure 2: Technical Drawing of the custom-built localizer

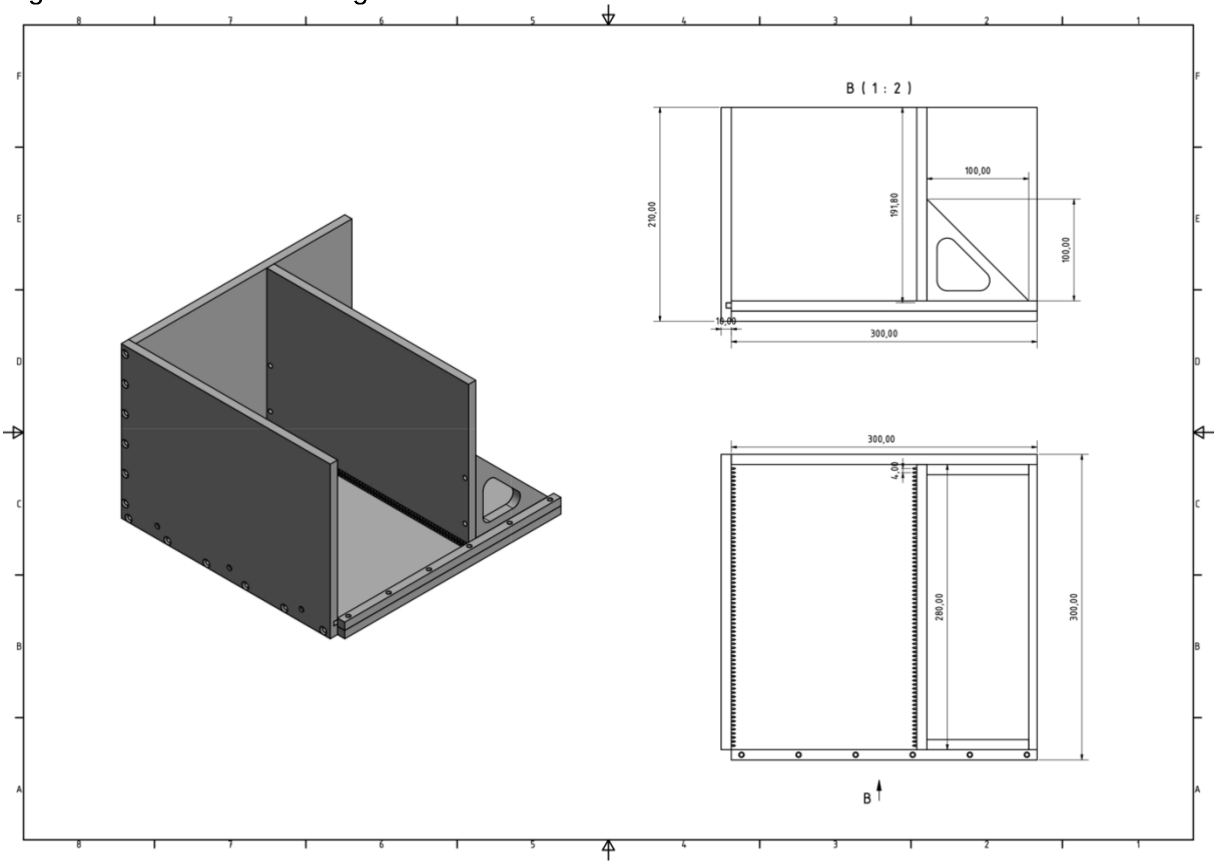

Note the variable side wall for different tumor sizes. A 4 mm measuring grid is attached to both sidewalls that is visible on MRI as a negative in the agarose block.
Standard for the exchange of product model data (STEP) file is provided online as supplemental data.

Figure 3: Technical Drawing of the custom-built cutting device

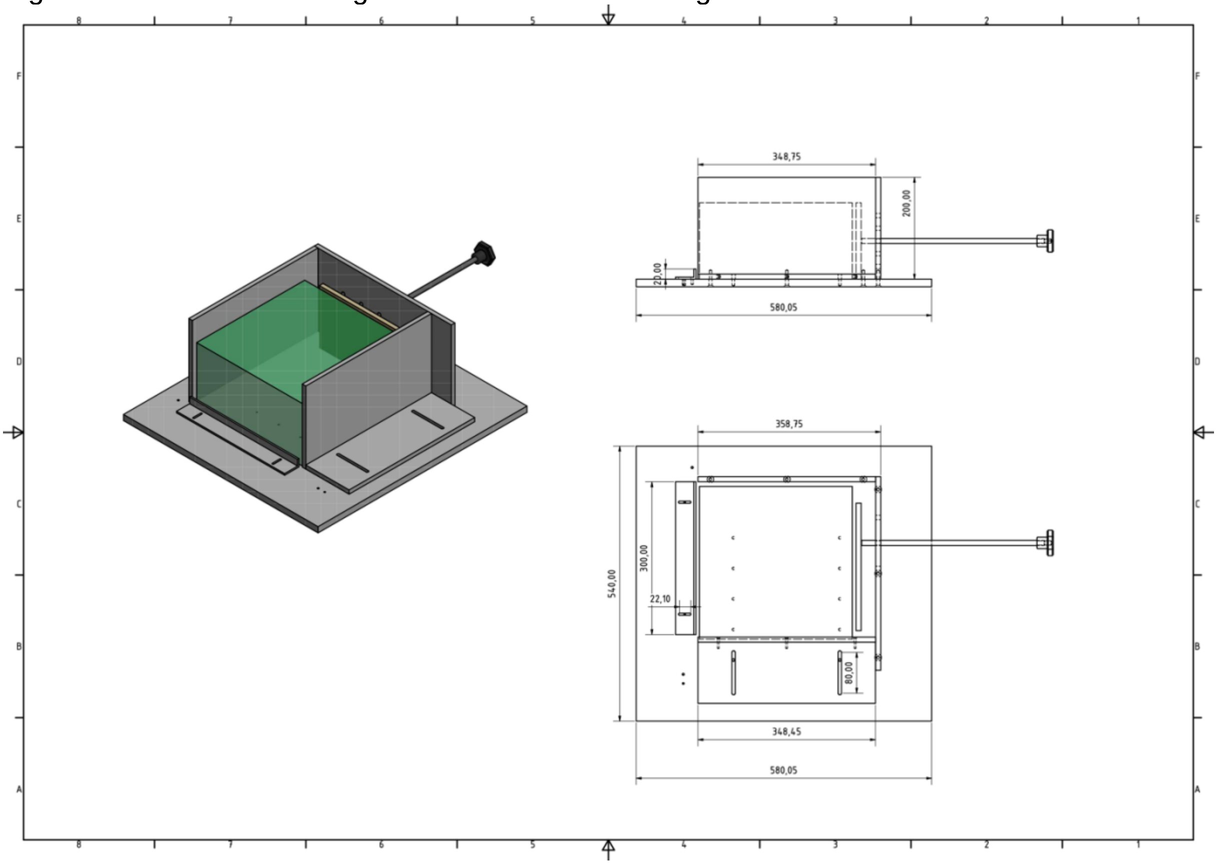

Note the variable side wall for different tumor sizes. The adjustable rail on the open side of the device allows the pathological sectioning of the specimen every 4 mm according to the sections of the second *ex vivo* MRI that was specified by the localizer (Supplemental Figure 2). Standard for the exchange of product model data (STEP) file is provided online as supplemental data.

Figure 4: Illustrative relationship of TRE to spatial scale of STS heterogeneity

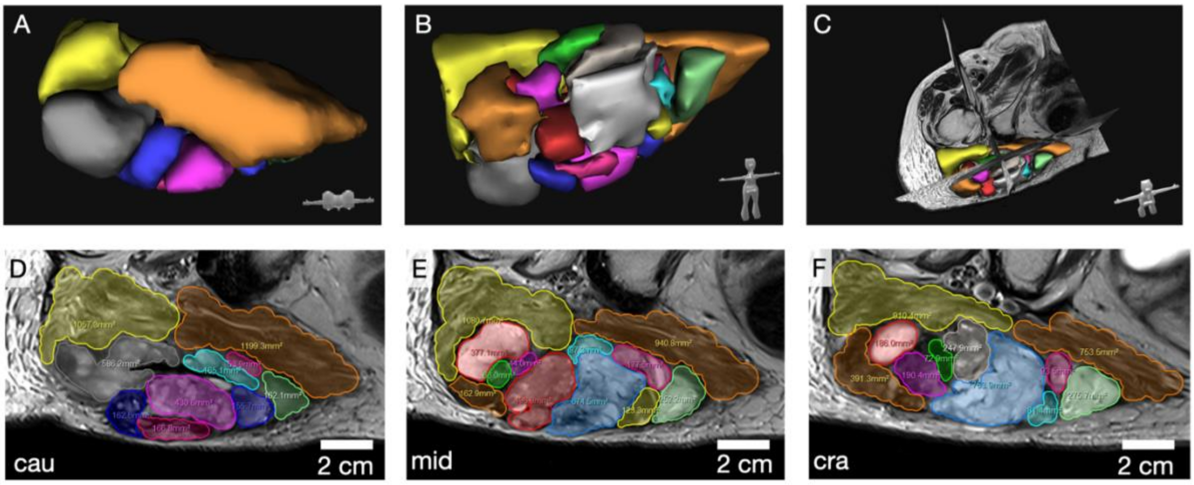

Exemplary illustrations of Patient 1. The volumes of the distinct tumor parts ranged from 0.8 to 65.6 mL (mean tumor volume ± SD: 11.88 ± 16.91 mL) with corresponding maximum axial diameters ranging from 10.2 to 39.9 mm. Our co-registration algorithm resulted in a mean TRE per STS volume of only 0.013 mm/mL in this patient. Therefore, all tumor subregions can be reliably identified.
